# The first complete mitochondrial genome sequence of *Cynopterusbrachyotis* (Chiroptera, Pteropodidae) from the Philippines

**DOI:** 10.3897/BDJ.10.e72768

**Published:** 2022-02-14

**Authors:** Paul Lorenzo A. Gaite, Wilson F. Aala, Jr., Michael G. Bacus, Christian C. Labrador, April Mae M. Numeron, Lief Erikson D. Gamalo, Lyre Anni E. Murao

**Affiliations:** 1 Philippine Genome Center Mindanao, University of the Philippines Mindanao, Davao City, Philippines Philippine Genome Center Mindanao, University of the Philippines Mindanao Davao City Philippines; 2 Department of Biological Sciences and Environmental Studies, University of the Philippines Mindanao, Davao City, Philippines Department of Biological Sciences and Environmental Studies, University of the Philippines Mindanao Davao City Philippines; 3 Wildlife-Human Interaction Studies, Ecological Research and Biodiversity Conservation Laboratory, University of the Philippines Mindanao, Davao City, Philippines Wildlife-Human Interaction Studies, Ecological Research and Biodiversity Conservation Laboratory, University of the Philippines Mindanao Davao City Philippines

**Keywords:** NGS, mitogenome, bats, taxonomy, phylogenetic analysis

## Abstract

The technical limitations of capillary sequencing in providing insights on phylogeny have been greatly aided in recent years by the implementation of next generation sequencing platforms which can generate whole mitochondrial genome (mitogenome) sequences. In this study, enriched mitochondrial DNA of *Cynopterusbrachyotis* from Mindanao, Philippines was sequenced using the Illumina MiSeq platform. A total of 653,967 clean paired-end reads was assembled using a MIRA-MITObim pipeline, resulting in a consensus mitogenome sequence length of 17,382 bases and a GC content of 41.48%, which is consistent with other published mitogenomes in fruit bats. The assembled *C.brachyotis* mitogenome was annotated using the MITOS online server and was able to resolve all mitochondrial genes, except for one transfer RNA gene (trnT) which may be further resolved by additional capillary sequencing of the region. Sequence analysis showed that the Philippine *C.brachyotis* is only 90%-91% homologous with other *Cynopterus* spp., based on its full mitogenome sequence. Phylogenetic analysis of fruit bat mitogenomes, deposited in online repositories, revealed that the Philippine *C.brachyotis* in this study has diverged from Asian *Cynopterus*, namely *Cynopterusbrachyotis* and *Cynopterussphinx* from other parts of Asia (100% bootstrap support) with the latter two forming a separate clade. This divergence at the species level was consistent with phylogentic inference using cytochrome oxidase 1 (CO1) and cytochrome B (cytb) gene markers. Our results strengthen the previously reported hypothesis that the Cynopteruscf.brachyotis in the Philippines is distinct from its Asian counterparts and should be, therefore, treated as a new species.

## Introduction

*Cynopterusbrachyotis* (Müller, 1838) is a fruit bat widespread in Southeast Asia, ranging from Sri Lanka, Taiwan, Vietnam, Indonesia, Malaysia and the Philippines (Jayaraj et al. 2012). The species is believed to have reached the Philippine islands within the past 1 million years (Rowe 2018). Currently, it is abundant in different types of habitats in the country with differing degrees of disturbances, ranging from urban residential, agricultural to forested areas (Rowe 2018). Due to high population abundance, widespread geographical extent and the relatively stable population, the species is considered of least concern under the updated IUCN Red List (Csorba et al. 2019, Rosell-Ambal et al. 2019).

The status of *C.brachyotis* taxonomy in the Philippines is uncertain and challenging. Genetic studies suggest that the Philippine population has high levels of divergence in comparison to other *C.brachyotis* populations in other Southeast Asian countries (Bacus et al. 2021, Luczon et al. 2019). Moreover, other authorities suggested that the Philippine *Cynopterus* should be assigned as *Cynopterusluzoniensis*, a taxon believed to exist throughout the Philippine archipelago and in the Sulawesi Island of Indonesia (Campbell et al. 2004, Rosell-Ambal et al. 2019, Rowe 2018, Simmons 2005). However, these hypotheses should be further evaluated as the exact identification of *C.luzoniensis* in Sulawesi may be different from "*C.luzoniensis*" in the Philippines or in some islands in the country (Campbell et al. 2004, Jayaraj et al. 2012). Due to this confusion on the distribution and extent of the taxa mentioned above, we will be consistently using "Cynopteruscf.brachyotis" in this paper to represent the Philippine *Cynopterus* to avoid confusion until taxonomy is resolved.

The analyses of mitochondrial genes are crucial to understand the challenging and confusing taxonomy and evolutionary history of bats in the Philippines. However, information on the mitochondrial genome (mitogenome) of Philippine bats is clearly fragmented despite the wide application of this data for nomenclature, evolution and phylogeny of many species. Fortunately, the technical limitations of capillary sequencing to provide phylogenetic evidence have been greatly aided by the use of Next Generation Sequencing (NGS) platforms that can generate whole mitogenome sequences.

As part of the larger initiative to sequence the mitochondrial DNA of native species in the country, we provide the complete mitochondrial genome (mitogenome) of Cynopteruscf.brachyotis from Mindanao Island. Moreover, phylogenetic trees, based on the sequences generated from this study and from those deposited in GenBank, were provided to show the evolutionary relationship of the Philippine population to other *Cynopterus* spp. from other countries in Asia. Results from this study will contribute to the taxonomic status and identity of the Philippine *Cynopterus* which, in turn, can be used as additional supporting information for other phylogenetic, evolution and ecological studies of bat species in the world.

## Material and methods

### Samples

The sample used in this study was a cardiac tissue from a voucher specimen, previously identified as *Cynopterusbrachyotis* using morphological characteristics and measurements (e.g. forearm length, head length, body length, tail length and ear length) with the aid of the taxonomic key developed by Ingle and Heaney (1992). Moreover, a ~ 202 bp barcode for the mitochondrial cytochrome oxidase 1 (CO1) gene (Walker et al. 2016) was determined in a separate study (Bacus et al. 2021) that further confirmed the identity of the specimen. An IACUC approval from the University of the Philippines Manila (Protocol No.: 2018-109) and a Gratuitous permit from the Philippine Department of Environment and Natural Resources Region XI (WGP No.: 2018-07) were obtained for the collection of voucher specimens.

### DNA Extraction and Bat Mitogenome Enrichment

Around 20 mg of cardiac tissue was obtained from a voucher specimen of *C.brachyotis*. Genomic DNA was extracted using the DNEasy Blood and Tissue kit (Qiagen, Hilden, Germany) according to the manufacturer’s instructions. Mitogenome enrichment was performed using two primer pairs, namely LR6F (5’-GCC CAT ACC CCG AAA ATG TTG-3’), LR6R (5’-CGG CGG GAG AAG TAG ATT GAA-3’), LR15F (5’-CCA CAG GAA AAT CAG CCC AAT T-3’) and LR15R (5'-GCT GTT GCT GTG TCT GAT GTG-3’) as previously described (Jebb et al. 2017). PCR amplicons were visualised with 0.7% agarose gel and stored at -20°C prior to the library preparation step.

### Library Preparation and Illumina Sequencing

The two PCR amplicons were quantified using Qubit dsDNA HS assay and normalised in a resuspension buffer to a final volume of 2.5 µl and a final concentration of 10 ng/µl (25 ng total yield). Both amplicons were pooled in a new PCR tube. DNA tagmentation, amplification and library clean-up were performed using the Nextera XT Library Prep Kit according to the manufacturer’s instructions. Cleaned libraries were then sent to the Philippine Genome Center Diliman-DNA Sequencing Core Facility for normalisation and next-generation sequencing using the Illumina MiSeq platform and a paired-end read format at 2 x 150 bp for 500 cycles.

### Mitogenome Assembly and Analysis

Raw sequencing reads were subjected to initial quality assessment with the FastQC software (Andrews 2010). Raw reads were trimmed of adapters and low-quality bases at both ends using Trimmomatic (Bolger et al. 2014). The filtered reads were used to generate an initial mapping assembly using MIRA (Chevreux et al. 2013) and were iteratively mapped back to the initial mapping assembly and assembled using MITObim (Hahn et al. 2013). The reference sequence that was used for the assembly was the NCBI Reference Sequence entry for *Cynopterusbrachyotis* mitochondrion, complete genome (GenBank Accession ID: NC_026465). The different genes/regions in the resulting assembly were annotated using the MITOS web server (Bernt et al. 2013). Both the entire assembled genome and the identified genes/regions were annotated individually using the nucleotide BLAST tool at the NCBI website (NCBI Resource Coordinators et al. 2018). Default settings were used in the parameters for running all of the aforementioned procedures. The assembled mitogenome sequence was submitted to NCBI GenBank and subsequently assigned the accession number OK163841.

### Phylogenetic Analysis

The assembled mitogenome sequence of *Cynopterusbrachyotis* from Mindanao, Philippines was aligned with available fruit bat mitogenome sequences in the NCBI GenBank. The best phylogenetic model was determined using MEGA 7, based on the lowest BIC and AIC values (Kumar et al. 2016). A phylogenetic tree was subsequently inferred using the GTR+G+I DNA substitution and site heterogeneity model using a Maximum-Likelihood estimation with 1000 bootstrap replicates in MEGA 7 (Kumar et al. 2016). A similar analysis was performed for the full cytochrome oxidase 1 (CO1) and cytochrome B (cytb) genes. For the CO1 gene, a multiple sequence alignment of 1,533 bp was used for the phylogenetic analysis using the Maximum-Likelihood method with the HKY+G+I model in MEGA 7 with 1000 bootstrap replicates. For the cytb gene, a multiple sequence alignment of 1,134 bp was used for the phylogenetic analysis using the Maximum-Likelihood method with the GTR+G+I model in MEGA 7 with 1000 bootstrap replicates. A distant vertebrate group, fish, was selected as the outgroup.

## Results

### Mitogenome

A total of 2,180,947 reads was initially generated by next generation sequencing of the mitochondrial DNA-enriched tissue sample (Table 1). The quality control steps reduced this number of reads to 1,784,834. From this readpool, a total of 653,967 reads was used for the assembly of the enriched mitochondrial DNA by the MIRA-MITObim workflow. The generated mitochondrial DNA assembly had a total length of 17,382 bases with a GC content of 41.48%. The average depth of coverage of this assembly was calculated to be 10,158.30.

**Figure 1.**
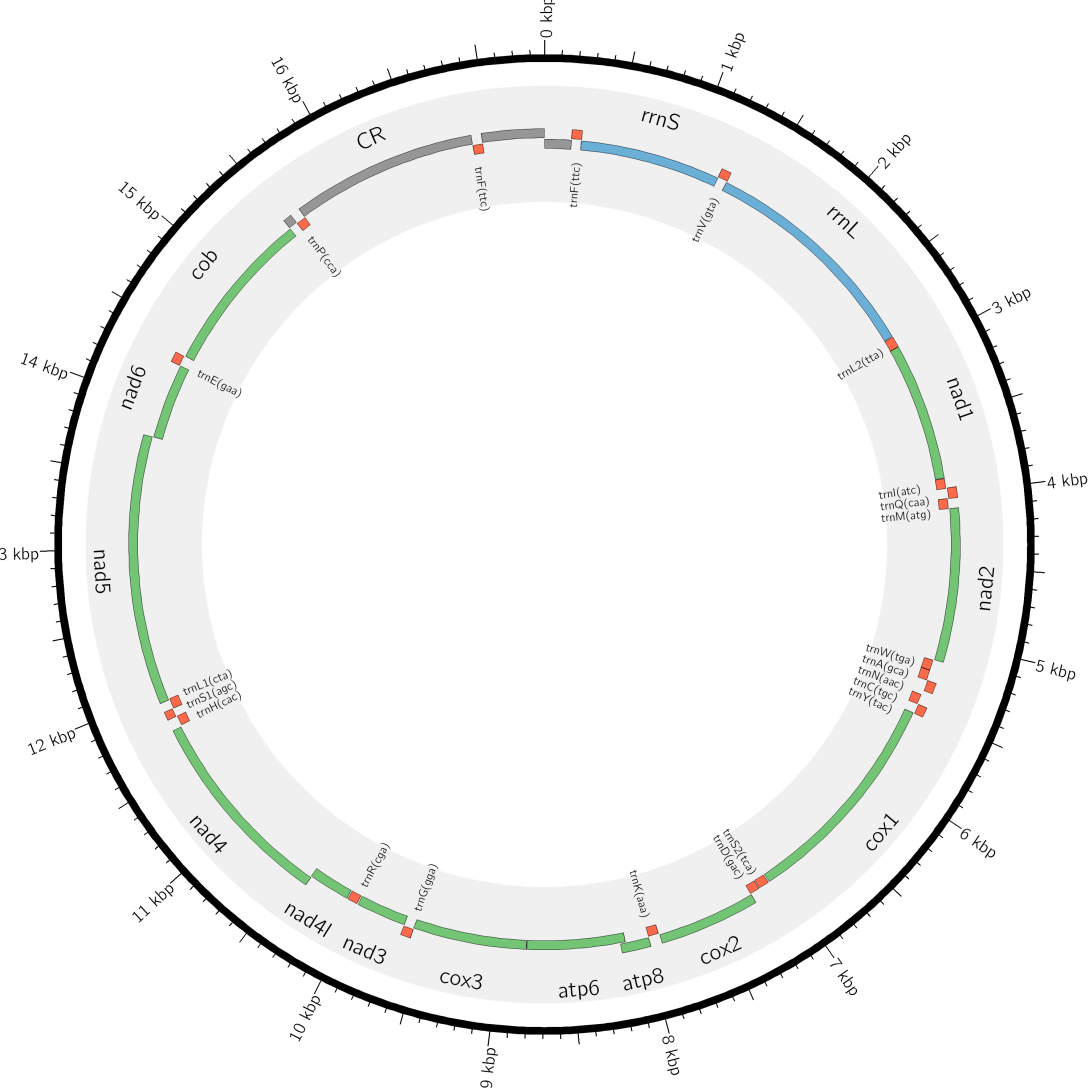
Graphical representation of the assembled Cynopteruscf.brachyotis mitochondrial genome with MITOS-annotated genes (figure generated with Circos software by Krzywinski et al. 2009).

The assembled sequence was subjected to online nucleotide BLAST search, with the top two hits being *Cynopterussphinx* voucher MZF1967 mitochondrion, complete genome (90.86% identity, 96% query coverage, 82 sequence gaps) and *Cynopterusbrachyotis* mitochondrion, complete genome (90.56% identity, 95% query coverage, 83 sequence gaps) (Table 2). Further annotation of the assembled sequence by MITOS identified a total of 36 genes: 21 tRNA genes, 2 rRNA genes and 13 protein-coding genes (Fig. 1). All known vertebrate mitochondrial genes have been found in this mitogenome, except trnT. Performing online nucleotide BLAST on individual gene regions identified by MITOS resulted in mostly either *C.brachyotis* or *C.sphinx* top hits with percent identities within the range of 90%-97%. It is also noted that the top hit for trnM(atg) has no significant similarity found with any NCBI-BLAST database entry, a few top hits are non-*Cynopterus* related entries (e.g. *Megaeropsniphanae* and *Myonycteristorquata*) and the top hits for trnY(tac) and trnD(gac) are non-bat related entries, such as *Maxomyssurifer* and *Equusasinus*, respectively (Table 3).

### Phylogenetics

**Figure 2.**
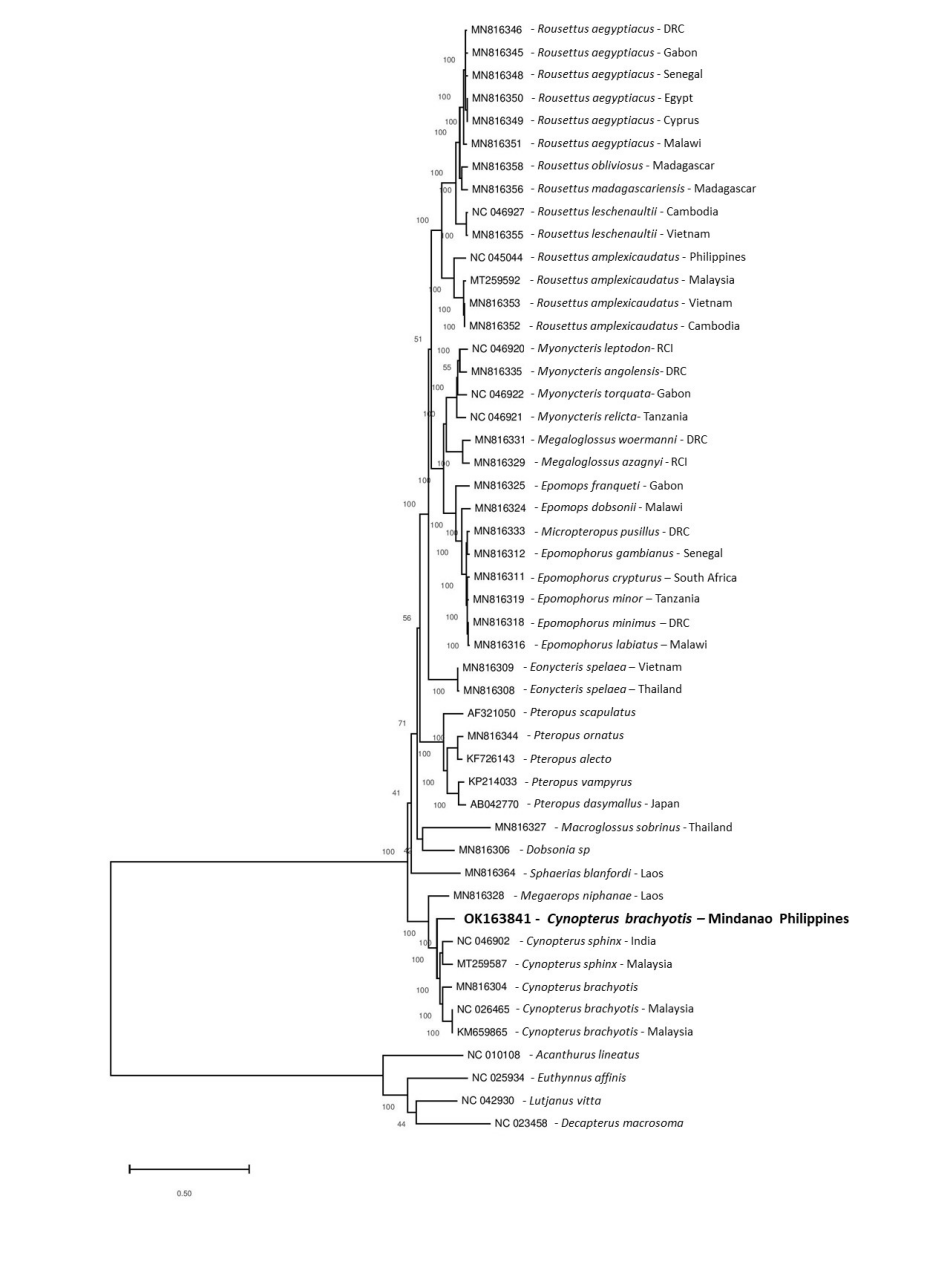
Maximum Likelihood phylogeny of selected fruit bat mitogenomes obtained from GenBank. The tree was generated using MEGA 7 with 1000 bootstrap replicates. The DNA substitution model used was GTR+G+I. The scale bar denotes the number of substitutions per site which is reflected in the branch lengths (i.e. 0.5 value means 5 nucleotide changes for every 10 nucleotides). Bootstrap values for clade support are written on the nodes of the branches. An outgroup consisting of fish mitogenomes was included in the phylogenetic tree inference.

**Figure 3.**
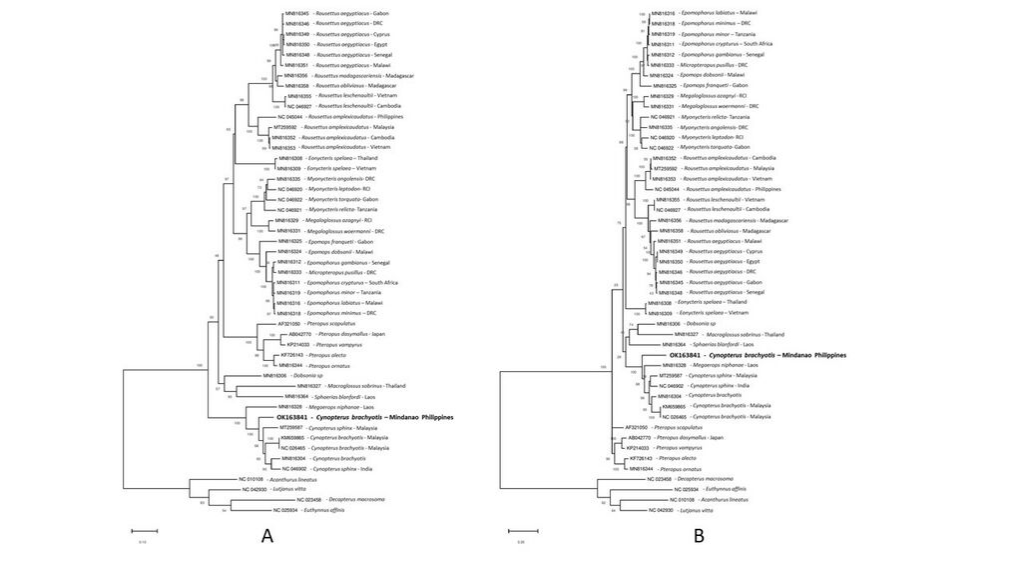
Maximum Likelihood phylogeny of fruit bat A) CO1 and B) cytb gene obtained from GenBank. The tree was generated using MEGA 7 with 1000 bootstrap replicates. For the CO1 gene, a multiple sequence alignment of 1,533 bp was used for the phylogenetic analysis using the Maximum-Likelihood method with the HKY+G+I model in MEGA 7 with 1000 bootstrap replicates. For the cytb gene, a multiple sequence alignment of 1,134 bp was used for the phylogenetic analysis using the Maximum-Likelihood method with the GTR+G+I model in MEGA 7 with 1000 bootstrap replicates. The scale bar denotes the number of substitutions per site which is reflected in the branch lengths (i.e. 0.5 value means 5 nucleotide changes for every 10 nucleotides). Bootstrap values for clade support are written on the nodes of the branches. An outgroup consisting of fish mitogenomes was included in the phylogenetic tree inference.

A phylogenetic tree, based on the complete mitogenome sequences of selected fruit bats, was generated (Fig. 2). The cynopterine fruit bats formed a monophyletic clade with 100% bootstrap support. Interestingly, the *Cynopterusbrachyotis* from Mindanao, Philippines appeared to have diverged from the rest of the Asian *Cynopterus* (*C.brachyotis* & *C.sphinx*) with a robust bootstrap support (100%). This branching pattern also appeared to be consistent with phylogenetic trees generated using the two commonly-used markers, CO1 and cytb genes (Fig. 3).

## Discussion

An almost complete mitogenome (99.86%, contains a single 25-base "N" gap in the final assembled sequence) was generated using an Illumina NGS and MIRA-MITObim assembly workflow. Nucleotide BLAST of the assembled mitogenome and individual genes confirmed its identity to be *Cynopterus* sp., the first reported mitogenome of this genus from the Philippines. Further annotation of individual mitochondrial genes by MITOS determined that almost all vertebrate mitochondrial genes were present on the assembled mitogenome, except for trnT. The length (17,382 bases) and GC content (41.48%) of the assembled mitogenome were similar and hence concordant with other published fruit bat mitogenomes (Jahari et al. 2020, Jahari et al. 2021, Yue 2019). These steps and resulting statistics confirm that a true, high-quality bat mitogenome has been assembled from the sequence reads and the identity of this assembled mitogenome can be attributed to *Cynopterus* sp.. However, the relatively low similarity of the whole mitogenome (90.86% and 90.56% to *C.sphinx* and *C.brachyotis*, respectively) and individual gene regions (90%-97%) to both *C.brachyotis* and *C.sphinx* entries suggest that the assembled mitogenome originated from a species that is genetically unique from the aforementioned. The presence of non-*Cynopterus* and non-bat BLAST hit results and even a hit result with no significant similarity, for a number of the mitochondrial genes due to low similarity to *Cynopterus*-related database entries, provide further evidence to this proposition.

Phylogenetic analysis of the mitogenomes and individual CO1 and cytb genes confirmed that the sequenced bat in this study formed a genetically-distinct clade from the Asian *C.brachyotis* and *C.sphinx*. Similar findings have been reported in recent years for the Philippine Cynopteruscf.brachyotis through sequence analysis of the standard (500 bp) and even shorter (200 bp) CO1, cytb and control region (Bacus et al. 2021, Campbell et al. 2004, Luczon et al. 2019). In fact, the standard CO1 barcode depicted a high genetic distance (11.5%) between the Philippine and Southeast Asian *C.brachyotis* (Luczon et al. 2019), supporting its classification as a distinct species (Francis et al. 2010). Confirmation of these findings through our mitogenome analysis further strengthens this proposition.

*Cynopterusbrachyotis* is hypothesised to be a species complex, based on sequence analysis of the mitochondrial cytb and control region sequences (Campbell et al. 2004). In the Philippines, two distinct lineages are present (Campbell et al. 2004), suggesting multiple colonisation events from multiple origins. The Sunda *C.brachyotis* lineage is present in Palawan Island and the rest of the Philippines form a distinct lineage which is proposed for taxonomic reassignment to "*C.luzoniensis*". However, this reassignment is not consistently accepted by some authors. Thus, we recommend further investigation using more genetic samples from different islands in the Philippines that includes representatives from the Sulawesi population to fully resolve the taxonomic issues revolving around the Philippine *Cynopterus*. Moreover, inconsistencies on the taxonomic identity involving this species complex suggests the importance of including morphological and ecological data in resolving taxonomic problems (Campbell et al. 2004).

Phylogenetic analysis, using both whole mitogenome and individual gene markers, such as CO1 and cytb, produced similar phylogenetic trees with high branch support values, suggesting that these gene markers can be used to reliably resolve taxonomic groups in fruit bats. While this holds true at the genus level, careful consideration should be given when using single gene markers for species delineation as exemplified by the discordance between the CO1 and cytb phylogenetic trees for the placement of *C.sphinx* within the cynopterine clade. Such contrasting branching patterns might arise especially when only a few representative samples are used in the analysis.

This study focused on the mitochondrial genome due to its utility in taxonomic resolution. Mitochondrial DNA evolves faster compared to nuclear genetic markers, generating a higher degree of sequence variation which makes it useful to resolve lower taxonomic levels for organisms (Chan et al. 2021, Hassanin et al. 2013). Furthermore, the relatively small size of the mitogenome (~ 16,000 base pairs long) makes it a practical alternative as a molecular marker. Despite these characteristics, the sole use of the mitochondrial genome in taxonomy remains to have certain limitations. Alternatively, nuclear markers represent neutral and paternal inheritance. In particular, single nucleotide polymorphisms (SNPs) and microsatellites exhibit high polymorphism amongst populations or individuals (Galbusera et al. 2000, Jiang et al. 2016). In addition, the highly-conserved nature of nuclear rRNA gene sequences makes it suitable for resolving higher taxonomic levels for organisms (Chan et al. 2021). Other studies have shown that combining mitochondrial and nuclear DNA analysis provided better taxonomic resolution (Abreu et al. 2020, Lambret-Frotté et al. 2012). A similar approach can be used in future studies to provide a more comprehensive and accurate perspective to ascertain the taxonomic resolution of the Mindanao or Philippine Cynopteruscf.brachyotis.

## Conclusions

To date, the discussion on *Cynopterus* taxonomy in the Philippines is still fraught with conflicting evidence and, as a result, perspectives as well. Hence, this study was conducted so as to address part of these conflicts by attempting to provide further genetic evidence on Philippine *Cynopterus* through the sequencing of the mitogenome of *Cynopterusbrachyotis.* This study was, indeed, able to generate an assembled *Cynopterusbrachyotis* mitogenome from PCR-enriched bat tissue DNA, which was subsequently proven to be a high-quality and complete mitogenome sequence as determined through further quality assessment and annotation steps. The annotation steps revealed the relatively low similarity of the assembled mitogenome and individual genes with *C.brachyotis* and *C.sphinx* mitochondrial genomes and genes, respectively, suggesting that the mitogenome originated from a species that is genetically different to *C.brachyotis* (as originally identified); hence, phylogenetic analysis was performed for further confirmation. Phylogenetic analysis of whole mitogenome and CO1/cytb genes revealed and confirmed divergence of the Philippine Cynopteruscf.brachyotis from Asian *Cynopterus* (*C.brachyotis* and *C.sphinx*) with high branch support values. The results of this study altogether provide further evidence to confirm a previous proposition that Philippine Cynopteruscf.brachyotis is a unique species. Although part of the conflicting discussion on Philippine *Cynopterus* has been resolved by the results of this study, future studies to obtain nuclear marker/genome data, complementing the mitogenome data as well as morphological, behaviour and ecological data, are still needed so as to further establish the taxonomic resolution and the geographical extent of the Philippine Cynopteruscf.brachyotis.

## Figures and Tables

**Table 1.** Summary of mitochondrial genome assembly statistics.

Total number of generated raw sequencing reads	2,180,947
Total number of reads after quality control processing	1,784,834
Total number of reads used for mitogenome assembly	653,967
Total length of assembled mitogenome sequence	17,382
% GC of assembled mitogenome sequence	41.48%
Average depth of coverage of assembled mitogenome	10,158.30

**Table 2.** Top five NCBI-BLAST hits of the assembled mitogenome sequence.

BLAST hit description	query cover	e-value	% identity	accession ID
*Cynopterussphinx* voucher MZF1967 mitochondrion, complete genome	96%	0	90.86	MT259587
*Cynopterusbrachyotis* mitochondrion, complete genome	95%	0	90.56	KM659865
*Cynopterusbrachyotis* isolate 140810x2 mitochondrion, complete genome	95%	0	91.29	MN816304
*Cynopterussphinx* isolate CKM35 mitochondrion, complete genome	96%	0	90.94	NC_046902
*Megaeropsniphanae* isolate NLN25 mitochondrion, complete genome	96%	0	88.27	NC_046915

**Table 3.** Top NCBI-BLAST hits for the MITOS-annotated mitochondrial genes.

mitochondrial gene (MITOS)	top hit annotation	query cover	e-value	% identity	accession ID
trnF(ttc)	*Cynopterussphinx* voucher MZF1967 mitochondrion, complete genome	100%	2.00E-22	97.1	MT259587.1
rrnS	*Cynopterussphinx* voucher MZF1967 mitochondrion, complete genome	99%	0	94.21	MT259587.1
trnV	*Cynopterussphinx* voucher MZF1967 mitochondrion, complete genome	100%	8.00E-26	100	MT259587.1
rrnL	*Cynopterusbrachyotis* isolate 140810x2 mitochondrion, complete genome	100%	0	96.31	MN816304.1
trnL2(tta)	*Cynopterussphinx* voucher MZF1967 mitochondrion, complete genome	96%	6.00E-28	100	MT259587.1
nad1	*Cynopterussphinx* mitochondrial ND1 gene for NADH dehydrogenase subunit 1, complete cds	100%	0	91.27	AB079802.1
trnI(atc)	*Megaeropsniphanae* isolate NLN25 mitochondrion, complete genome	100%	1.00E-24	98.55	NC_046915.1
trnQ(caa)	*Cynopterussphinx* voucher MZF1967 mitochondrion, complete genome	100%	2.00E-23	95.89	MT259587.1
trnM(atg)	(no significant similarity found)	-	-	-	-
nad2	*Cynopterusbrachyotis* isolate 140810x2 mitochondrion, complete genome	97%	0	89.33	MN816304.1
trnW(tga)	*Cynopterusbrachyotis* isolate 140810x2 mitochondrion, complete genome	100%	1.00E-23	97.14	MN816304.1
trnA(gca)	*Myonycteristorquata* isolate 1732 mitochondrion, complete genome	100%	2.00E-26	100	NC_046922.1
trnN(aac)	*Cynopterussphinx* isolate CKM35 mitochondrion, complete genome	100%	2.00E-18	91.78	NC_046902.1
trnC(tgc)	*Cynopterussphinx* isolate CKM35 mitochondrion, complete genome	98%	9.00E-21	95.59	NC_046902.1
trnY(tac)	*Maxomyssurifer* voucher CBGP_R4223 mitochondrion, complete genome	100%	5.00E-23	98.51	KY464181.1
cox1	*Cynopterusbrachyotis* mitochondrion, complete genome	100%	0	91	KM659865.1
trnS2(tca)	*Cynopterussphinx* voucher MZF1967 mitochondrion, complete genome	100%	2.00E-26	100	MT259587.1
trnD(gac)	*Equusasinus* isolate 9348 mitochondrion, complete genome	98%	1.00E-24	100	MK896308.1
cox2	*Cynopterusbrachyotis* isolate 140810x2 mitochondrion, complete genome	100%	0	91.48	MN816304.1
trnK(aaa)	*Rousettusamplexicaudatus* voucher MRMD 2101 mitochondrion, complete genome	100%	4.00E-19	92.96	NC_045044.1
atp8	*Cynopterusbrachyotis* mitochondrion, complete genome	100%	2.00E-77	94.36	KM659865.1
atp6	*Cynopterusbrachyotis* mitochondrion, complete genome	100%	0	90.96	KM659865.1
cox3	*Cynopterusbrachyotis* isolate 140810x2 mitochondrion, complete genome	100%	0	93.1	MN816304.1
trnG(gga)	*Megaeropsniphanae* isolate NLN25 mitochondrion, complete genome	100%	2.00E-22	97.06	NC_046915.1
nad3	*Cynopterussphinx* voucher MZF1967 mitochondrion, complete genome	100%	8.00E-129	91.3	MT259587.1
trnR(cga)	*Megaeropsniphanae* isolate NLN25 mitochondrion, complete genome	100%	4.00E-24	98.55	NC_046915.1
nad4l	*Cynopterusbrachyotis* mitochondrion, complete genome	100%	1.00E-105	91.58	KM659865.1
nad4	*Cynopterussphinx* isolate CKM35 mitochondrion, complete genome	100%	0	92.25	NC_046902.1
trnH(cac)	*Cynopterussphinx* voucher MZF1967 mitochondrion, complete genome	100%	4.00E-24	98.53	MT259587.1
trnS1(agc)	*Cynopterussphinx* voucher MZF1967 mitochondrion, complete genome	100%	6.00E-21	100	MT259587.1
trnL1(cta)	*Rousettusamplexicaudatus* voucher MZF1072 mitochondrion, complete genome	100%	7.00E-27	100	MT259592.1
nad5	*Cynopterusbrachyotis* isolate 140810x2 mitochondrion, complete genome	99%	0	89.88	MN816304.1
nad6	*Cynopterusbrachyotis* isolate 140810x2 mitochondrion, complete genome	100%	0	89.83	MN816304.1
trnE(gaa)	*Cynopterusbrachyotis* mitochondrion, complete genome	100%	2.00E-21	95.65	KM659865.1
cob	*Cynopterusbrachyotis* mitochondrial cytb gene for cytochrome b, complete cds, country: Philippines: Davao del Norte, Panabo, Tibungol	100%	0	87.59	AB046321.1
trnP(cca)	*Megaeropsniphanae* isolate NLN25 mitochondrion, complete genome	100%	1.00E-19	95.52	NC_046915.1
trnF(ttc)	*Cynopterussphinx* voucher MZF1967 mitochondrion, complete genome	100%	2.00E-22	97.1	MT259587.1

## Hosting institution

Philippine Genome Center Mindanao, University of the Philippines Mindanao, Davao City, Philippines
